# A Comparative Study on Characteristics and Antibacterial Capacity of Cotton Fabrics Dyed with Reactive Dye and *Diospyros Mollis* Extract

**DOI:** 10.1002/open.202400130

**Published:** 2024-07-31

**Authors:** Trong Tuan Nguyen, Thuy Chinh Nguyen, Thi Thu Trang Nguyen, Manh Ha Nguyen, Hoang Thai

**Affiliations:** ^1^ Graduate University of Science and Technology Vietnam Academy of Science and Technology 18 Hoang Quoc Viet Street, Cau Giay District Hanoi 100000 Vietnam; ^2^ Faculty of Garment Technology and Fashion Design Hanoi University of Industry 298 Cau Dien Street, Bac Tu Liem District Hanoi 100000 Vietnam; ^3^ Institute for Tropical Technology Vietnam Academy of Science and Technology 18 Hoang Quoc Viet Street, Cau Giay District Hanoi 100000 Vietnam

**Keywords:** Diospyros mollis (Griff) extract, Reactive dye, Textile dye, Natural dye, Textile dye wastewater

## Abstract

This article focuses on comparing the characteristics of cotton fabric dyed with *Diospyros mollis* extract (DME) solution and that of cotton fabric dyed with the reactive dye. The parameters of the cotton fabric after dyeing with both types of dyes were assessed, including color strength (K/S), structural morphology, infrared spectrum, antibacterial properties, UV resistance, color fastness to washing, rubbing, light, moisture absorption, breathability, and wastewater indices. The obtained results show that the K/S value of cotton fabric dyed with DME solution is slightly lower than that of cotton fabric dyed with the reactive dye, 18.52 and 19.36, respectively. The cotton fabric dyed with the reactive dye does not exhibit antibacterial activity against *Escherichia coli* and *Staphylococcus aureus*, whereas the antibacterial effectiveness against these bacteria for cotton fabric dyed with DME solution is 99.99 %. The UV protection capability of cotton fabric dyed with DME solution is superior to cotton fabric dyed with the reactive dye. The BOD/COD ratio of wastewater from the dyeing process with DME is higher than that of the reactive dye, with values of 0.70 and 0.32, respectively. The findings of this study indicate the superior ability of using DME solution as compared to the reactive dye, which is promising as a natural dye for fabric in medical applications.

## Graphical abstract

The cotton fabrics dyed with aqueous Diospyros mollis Griff extract (DME) has shown a high color fastness to washing, light, and rubbing, antibacterial ability against *E. coli* and *S. aureus* 99.9 %, great UV resistance efficiency, good air permeability, water vapor absorbance, a high tensile property as compared to the cotton fabrics dyed with Remazol deep black RGB active dye.

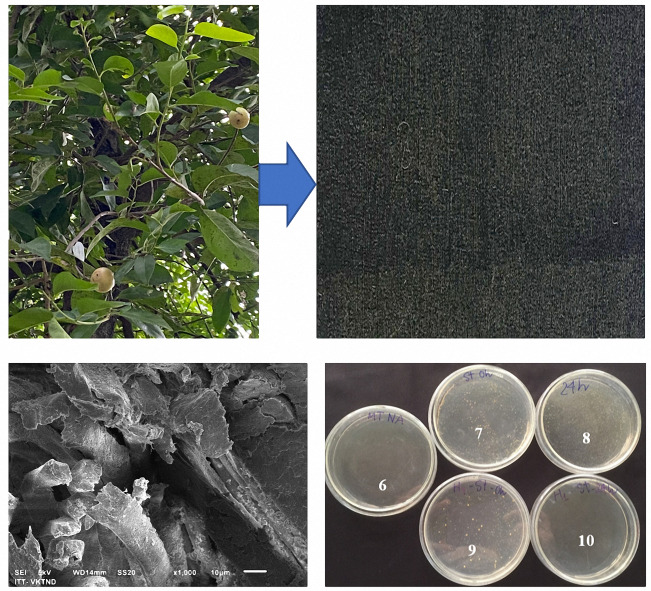

## 1 Introduction

The wastewater generated by the textile industry contains various pollutants such as organic compounds, unstable colorants, acids, alkalis, and notably highly toxic dyes.^[1]^ A portion of synthetic dyes fixed on the fabric and non‐fixed was discharged into the environment through wastewater, with an estimated amount ranging from 10 % to 50 % of the total dye used for fabric dyeing.^[2]^ Dye residues can enter the food chain, bioaccumulate, affect photosynthesis, and have the potential to cause harmful effects on the environment, leading to mutations and cancer if these untreated wastewater are released into the environment.^[3]^ In aquatic environments, dye pollution inhibits the growth of algae and hinders the transport of nutrients and energy of microorganisms in water.[^4^, ^5^] Textile dyes are extremely hazardous and contain carcinogenic aromatic chemicals.[^6^, ^7^, ^8^] They are associated with a range of disorders in both humans and animals, including skin inflammation and central nervous system issues.[^9^, ^10^, ^11^] Individuals working with active dyes are at risk of allergies, involving occupational asthma, allergic conjunctivitis, and contact dermatitis. The toxicity of textile dyes poses the greatest long‐term health risk to humans.[^12^, ^13^, ^14^]

In addition to synthetic dyes used for fabric dyeing, numerous studies have identified a rich source of natural materials from plants such as leaves, bark, roots, bulbs, fruits, and seeds that can be capable of dyeing fabrics. Some advantages of these natural materials are their excellent capacity for antimicrobial, antifungal, ultraviolet (UV) protection, insect‐repellent properties, and pleasant scents, attributed to a group of bioactive molecules known as phytochemicals. The primary chemicals in natural dyes include saponins, tannins, flavonoids, glycosides, and anthocyanins.^[15]^ Adeel *et al*. indicated that tea leaves dyed wool fabric in a natural reddish‐brown color with great antibacterial and antifungal properties.^[16]^ Cotton fabric dyed with mango tree extract has colorfastness grade 4–5.^[17]^ Pomegranate peel‐dyed cotton fabric has good colorfastness, lightfastness, antibacterial properties, and UV protection after 10 washes.^[18]^ Cotton fabric dyed with wood extracts from oak, indigo, and jujube fruit increases its tensile strength by 9.34, 5.69, 9.75, and 7.31 %, respectively. In addition, the biodegradability of natural dyes is over 93 %.^[19]^ *Conocarpus erectus* L. leaf extract used for dyeing wool and silk fabrics effectively produces colors with fabric color strength (K/S) values of 2.47 and 2.57, respectively. Moreover, the dyed fabrics exhibited high antibacterial activity against *S. aureus* and *E. coli*, over 81 %. The dyed fabrics achieved friction fastness grades 3–4 for dyed silk and 5–6 for dyed wool; lightfastness grades 4–5 for dyed silk and 5–6 for dyed wool; UV protection factor of 30 for dyed wool and 24 for dyed silk; and mosquito repellency of 36 % for dyed wool and 68 % for dyed silk.^[20]^ Alizarin extracted from *Rubia tinctorum* roots used for dyeing wool fabric has good washing color fastness, friction fastness grade 4–5, and antibacterial activity against *P. aeruginosa* and *E. coli* with inhibition zones of 23 and 15 mm, respectively.^[21]^ Organic cotton, viscose, and modal knitted fabrics dyed with annatto dye and chitosan finish achieve washing color fastness grade 4, 3–4, 3–4; friction fastness grade 4, 4, 3–4; sweat fastness grade 4, 3–4, 3–4; and lightfastness grade 4, 3–4, 3–4, respectively.^[22]^ *Eucalyptus globulus* leaf extract with high lignin content resists microbial and enzymatic activities, as well as possesses some medicinal properties, including anti‐inflammatory, anticancer, antibacterial, antifungal, and skin‐soothing effects.[^23^, ^24^, ^25^] When dyed cotton, the color of the fabric can range from yellow to brown depending on the concentration. Cotton fabric dyed with *Eucalyptus globulus* leaf extract achieves washing color fastness grade 3–4; friction fastness grade 4–5; and lightfastness grade 4.^[26]^ Antibacterial activity against *E. coli* and *S. aureus* of the fabrics reached 56.2 % to 88.5 % after 20 cycles of washing.[^27^, ^28^] The ultraviolet protection factor (UPF) of fabric after dyeing is 34 and increases to a value of 60 in the presence of chitosan.[^29^, ^30^] Indigo‐dyed cotton fabric has washing color fastness grade 3–4; light fastness grade 4–5;[^31^, ^32^] antibacterial ability against two *E. coli* and *S. aureus* bacterial strains over 83 %; and resistance to UV rays for indigo‐dyed cotton fabric is 36–38 while UV resistance for indigo dyed polyester fabric is 198.40.[^33^, ^34^, ^35^]

*Diospyros mollis* (Griff) trees are distributed in countries of Southeast Asia. The *Diospyros mollis* extract (DME) is used for dyeing silk, polyamide, cotton, oilcloth, and wool fabrics.[^36^, ^37^, ^38^, ^39^, ^40^] The main substance that makes DME activate as a green dye is diospyrol, 8,8′‐di‐ O ‐( β‐D‐glucopyranoside), which accounts for about 2 %. The color of fabrics dyed with DME can vary from light gray to black.[^36^, ^37^] Fabrics dyed with DME exhibit many desirable properties, such as high antibacterial ability, good UV resistance, high color fastness to washing, light, and rubbing, and increased moisture absorption and breathability. Polyamide fabrics have high color fastness and achieve colors ranging from gray to brown when dyed with DME under optimal conditions including extract/water ratio of 1/3.5 (v/v), dyeing temperature of 90 °C, and dyeing time of 60 minutes.^[36]^ Recently, natural dye colors based on DME extracted with diethyl ether, acetone, ethanol, and distilled water have been prepared for dyeing silk fabrics. The DME in acetone exhibits a positive effect on the color fastness of silk fabrics.^[37]^ Additionally, the DME in water can significantly increase the color strength, color fastness, antibacterial activity against both *Staphylococcus aureus* and *Escherichia coli*, and ultraviolet protection of wool fabrics.[^38^, ^39^]

In our previous study, the DME in water was used for dyeing cotton fabrics.^[40]^ The optimal conditions for the dyeing process have been found at a dyeing temperature of 56.36 °C, dyeing time of 90 minutes, and extraction ratio/water of 89/100 (v/v). The dyed cotton fabrics exhibit well antibacterial activity with the inhibition percentage for *Escherichia coli* and *Staphylococcus aureus* above 99 %. Some characteristics of dyed cotton fabrics have been assessed including color change, color strength, breathability, moisture absorption, thermal stability, and morphology. However, the UV resistance ability and tensile strength of dyed cotton fabrics have still not been evaluated. Additionally, the quality of wastewater after the dyeing process has been limited in the report. Moreover, the comparison of DME with the active dye is necessary to give an overview of the advantages of DME, a natural dye, for dyeing various types of fabrics.

There are different methods of dyeing fabrics, such as batik, cross‐dyeing, piece dyeing, vat dyeing, yarn dyeing, solution dyeing, etc. with some modern dyeing techniques, including ultrasonic technique, plasma technology, air dyeing, etc..[^41^, ^42^] Among them, solution dyeing is one of the most useful methods for dyeing cotton fabrics with a bright, clear appearance of products and the dyeing time is shortened.

This study focused on comparing the properties of the cotton fabrics dyed with DME in water with the cotton fabrics dyed with an active dye (Remazol midnight black – RGB), including structural morphology, antibacterial properties, UV resistance, breathability, moisture absorption, tensile strength, elongation at break, colorfastness to washing, rubbing, light, etc. This aims to assess the dyeing efficiency and ability to replace synthetic dyes of the DM extract. The DM fruits are available in our country, low cost, safe, and friendly while RGB is an active dye, produced from chemicals and additives, causes harm to human skin, and very difficult to biodegrade. The findings are basic to demonstrate the superior effectiveness of DME in fabric dyeing. Moreover, this study focused on the comparison of DME and RGB to provide direction for the development of traditional fabric dyeing villages in Vietnam. Additionally, the study evaluates the indicators of dye wastewater eliminated from two dyeing processes, with DME and with the active dye, such as BOD_5_ (biochemical oxygen demand), COD (chemical oxygen demand), TDS (total dissolved solids), TSS (total suspended solids), pH value, and indicators of dyed cotton fabric to assess the “green” effect of the natural DME dye.

## Experimental

### Materials

The woven cotton fabrics were produced by Bao Minh Vietnam Ltd. with a twill weave pattern, a warp yarn density of 102 yarns/inch, and a weft yarn density of 66 yarns/inch, both warp and weft yarns are carefully combed cotton with a count of 40, and the cotton fabric is pre‐treated to remove impurities.^[40]^

**Figure 1:**
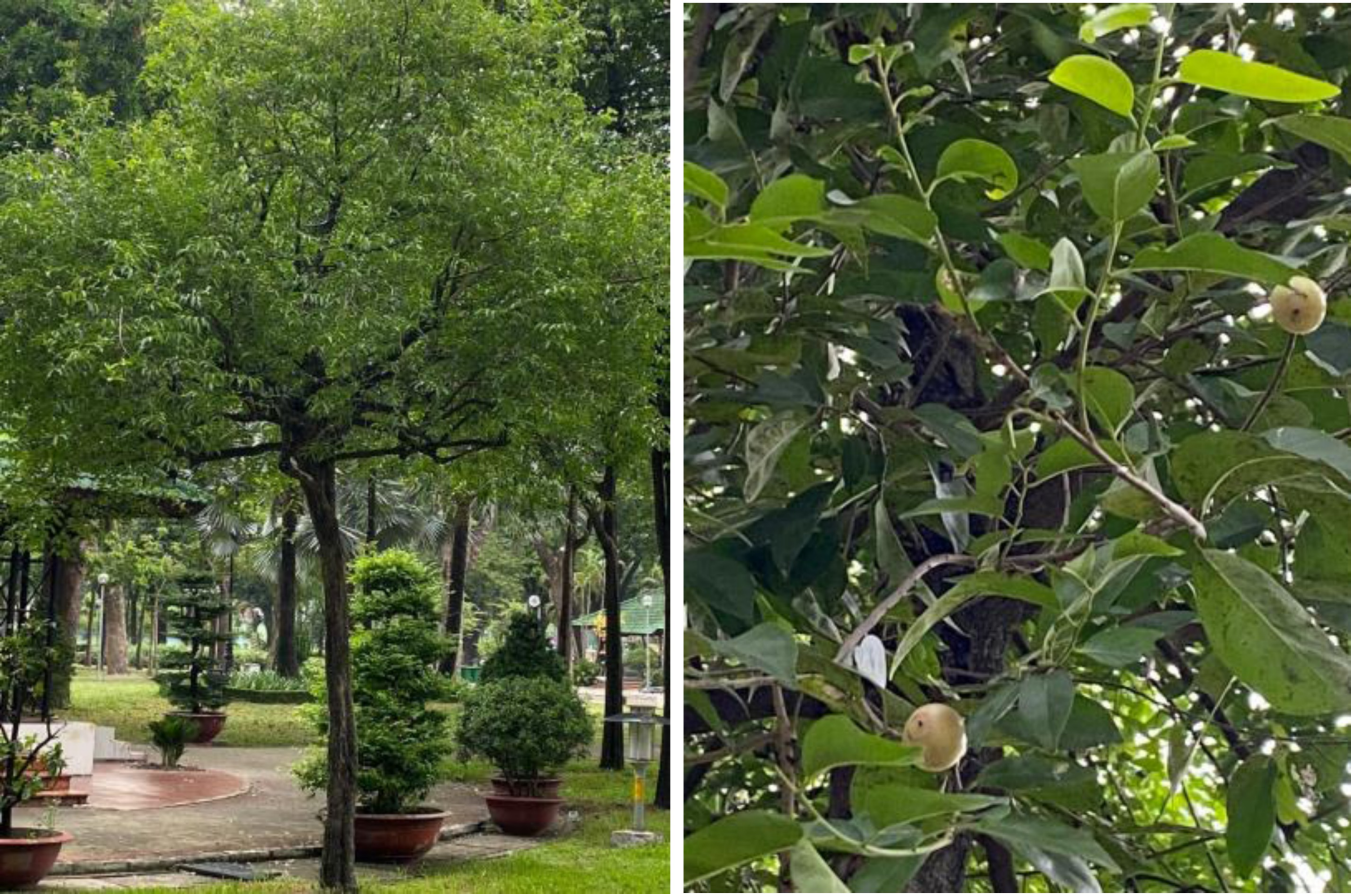
Diospyros mollis tree and fruits. The photos were taken at Gia Dinh Park, Ho Chi Minh City, Vietnam.

*Diospyros mollis* fresh fruits were sourced from An Giang province, Vietnam. Figure 1 presents the photos of *Diospyros mollis* trees and fruits. They were harvested in May 2023 and preserved at 5 °C without direct exposure to light and atmospheric oxygen. DME was prepared according to the procedure reported in the previous paper.^[40]^ The active dye, Remazol midnight black RGB, in a powder form, black color was provided by Aladdin Co., China. The auxiliary chemicals such as Sera Lube M–CF wrinkle resistant, Sera‐Wet C‐NR wetting agent, and Sera Quest C‐USP color leveling agent were purchased from Dyestar Chemicals Ltd., Soda (China). The laundry soap for washing cotton fabric is Standard soap (UK).

### Preparation and Characterization

#### Dyeing Process

**Figure 2:**
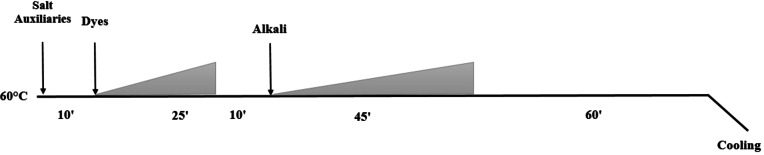
Procedure for dyeing process of cotton fabrics with the active dye Remazol Deep Black RGB

The process of dyeing the cotton fabrics with DME follows the procedure reported in the previous paper^[40]^ using a Ti‐Color I dye machine (Italy). The cotton fabrics were dyed three times with DME solution. For the dyeing process of the cotton fabrics with the active dye, firstly, the solutions of salt and dyeing auxiliaries (Sera Lube M‐CF, Sera‐Wet C‐NR, Sera Quest C‐USP; Remazol Deep Black RGB; and alkali solution (Soda) were prepared. Next, 10 g of the cotton fabrics was dyed on a Ti‐Color I dye machine (Italy) with the salt and auxiliary solution first, followed by the dye solution, and finally adding alkali solution into the dyeing machine. The dyed cotton fabrics were dried until a constant weight before continuing dyeing. The cotton fabrics were dyed with the active dye three times. The procedure for dyeing the cotton fabrics with the active dye is presented in Figure 2.

#### Dyeing Capacity of Cotton Fabrics

The color parameters of the dyed cotton fabrics were expressed according to CIELab coordinates (L*, a*, b*), in which, L* corresponds to brightness (100=white, 0=black); a* corresponds to red‐green coordinate; and b* corresponds to yellow‐green coordinate. The chroma, C*, represents the brightness or opacity and the color angle, H*, of samples were calculated using equations (1) and (2): 
(1)

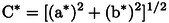

(2) $$H^{*}=\arctan\left(b^{*}/a^{*}\right)$$

The color change (ΔE*) of the dyed cotton fabric samples after dyeing and washing test was calculated according to equation 3:

(3) $$\Delta E^{*}=\sqrt{{\left(\Delta \alpha^{*}\right)}^{2}+{\left(\Delta b^{*}\right)}^{2}+{\left(\Delta L^{*}\right)}^{2}}$$

Where, ΔL^*^=L^*^_sample_−L^*^_control_, Δa^*^=a^*^_sample_−a^*^_control_, Δb^*^=b^*^_sample_−b^*^_control_.

The color strength (K/S) of the dyed cotton fabrics was calculated using Kubelka‐Munk equation:^[43]^

(4) $$\frac{K}{S}=\;\frac{{\left(1-R\right)}^{2}}{2R}$$

Where R is the reflectance of the sample at the maximum absorption wavelength ranging from 360 to 750 nm, K is the absorption coefficient and S is the scattering coefficient.

The color of samples was recorded using Xrite CI4200 colorimeter (USA) at room temperature (27±1 °C) with D65 light and an observation angle of 10°.

#### Analysis of Surface Morphology and Functional Groups of the Dyed Cotton Fabrics

The morphology of the dyed cotton fabrics was assessed by scanning electron microscopy (SEM) on a JSM‐6510LV (Jeol – Japan). The functional groups of the dyed cotton fabrics were evaluated through infrared spectroscopy (IR) using attenuated total reflection (ATR) technique in the wavenumbers ranging from 4000 to 400 cm^−1^, 32 scans and 8 cm^−1^ resolution on a Nicolet iS10 (Thermo Scientific, USA).

#### Assessment of Antibacterial Activity of the Dyed Cotton Fabrics

Antibacterial activity of dyed cotton fabrics against *Escherichia coli* and *Staphylococcus aureus* was determined according to AATCC 100‐2019 standard. Bacteria were cultured in nutrient environments, then the microbial solution was diluted at 37 °C to achieve a bacterial density of 1–3×10^5^ CFU/mL. Pieces of the cotton fabric samples that were cut into circular shapes with a diameter of 4.8 mm were placed in dishes containing the cultured bacteria. The bacterial density in the dishes was monitored after 24 hours of testing by counting colony‐forming units (CFUs). The percentage reduction of bacteria on the dyed cotton fabrics was calculated according to the formula:

(5) $$R=\frac{C-A\;}{C}100$$

Where A represents the number of CFUs in the cotton fabrics infected at 24 hours, B represents the number of CFUs in the cotton fabrics infected at 0 hour, and C represents the number of CFUs in the control sample at 24 hours.

#### Determination of UV Resistance of Dyed Cotton Fabrics

The UV‐resistant ability of the dyed cotton fabrics was evaluated based on the Ultraviolet Protection Factor (UPF) following the EN 13758‐1:2001 test method using a Labsphere spectrophotometer (USA). The UPF rating of a fabric material is a measure of how effectively it reduces UV radiation that can cause skin reddening. This rating is determined by conducting UPF tests across both the UVA and UVB regions. The UPF value for each fabric sample was calculated using the relative erythemal action spectrum (E_λ_), standard solar spectral irradiance (S_λ_), optical total transmittance of the fabric specimen (T_λ_), and the measured wavelength interval (Δ_λ_) according the equation (6).

(6) $$UPF=\frac{\sum_{280nm}^{400nm}E_{\lambda }S_{\lambda }\Delta \lambda }{\sum_{280nm}^{400nm}E_{\lambda }S_{\lambda }T_{\lambda }\Delta \lambda }$$

The average transmittance values in the UV>A and UV‐B regions, represented by *T*(*U*V‐*A*)_AV_ and *T*(*U*V‐B)_AV_ are crucial in determining the UPF rating. These values are expressed as equations (7) and (8), respectively.

(7) $$\;{T(UV-A)}_{AV}=\frac{\sum_{315nm}^{400nm}T_{\lambda }\Delta \lambda }{\sum_{315nm}^{400nm}\Delta \lambda }$$

(8) $$\;{T(UV-B)}_{AV}=\frac{\sum_{290nm}^{315nm}T_{\lambda }\Delta \lambda }{\sum_{290nm}^{315nm}\Delta \lambda }$$

#### Determination of Air Permeability of Dyed Cotton Fabrics

The air permeability of the cotton fabric samples was determined according to ISO 9237 : 1995 standard using an air permeability measuring device (Shirley, UK). The samples were conditioned in an environment with a temperature of 70±2 °F and a humidity of 65±2 %.

#### Determination of Water Vapor Absorption of Dyed Cotton Fabrics

The water vapor absorption of the cotton fabric samples was determined according to TCVN 5091 : 1990 standard. The cotton fabric samples were cut into 10×10 cm squares before conditioning them in an environment with a relative humidity of 100 %, and a temperature of 27±5 °C. After 4 hours of conditioning, the samples were weighed to determine the mass of the samples (m_i_). The samples were then dried at a temperature of 105 °C until a constant mass (m_a_) was reached. The water vapor absorption (H_w_) of the cotton fabric samples was calculated using the below formula: 
(9)

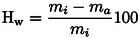




#### Tensile Strength and Elongation at Break of Dyed Cotton Fabrics

The tensile strength and elongation at the break of the dyed cotton fabric samples were performed on a Titan 10 tensile testing machine (UK) according to the TCVN 1754 standard. Three fabric samples with a size of 200×50 (mm) were prepared along the warp direction and three ones were prepared along the weft direction. The test was stored in a standard condition with a humidity of 65±2 %, and a temperature of 20±2 °C for 24 hours before the test.

#### Color Fastness to Washing, Rubbing, and Light of Dyed Cotton Fabrics

– Color fastness to washing of the fabric samples after 1, 10, 20, and 30 wash cycles was conducted according to the AATCC 61 standard. The washing process was carried out on a SW equipment (China), with the fabric samples sized 50×100 mm. Neutral soap (0.37 grams) was used along with 10 steel balls. Washing using 200 mL of water was carried out at 40 °C for 45 minutes. After washing, the fabric samples were rinsed with water at 30 °C.
– Color fastness to rubbing of the fabric samples was tested following the ISO 105‐D02 : 2016 standard on a Staining Tester (South Korea). The fabrics were rubbed according to the ISO 105‐F09 with a size of 50×140 mm. The solvent used was white spirit (China).
– Color fastness to light of the fabric samples was tested following the ISO 105‐B02 : 2014 standard on Xenotest Alpha LM equipment (USA), using a set of blue wool standards with increasing color fastness to light from 1 to 8. The fabric samples sized 70×120 mm were exposed to a 500‐watt mercury lamp for 5 to 640 hours. After light exposure, color fastness to light was evaluated using a gray scale according to the ISO 105‐A02 standard.

#### Determination of Dye Wastewater Characteristics

The characteristics of dye wastewater were determined, including biochemical oxygen demand (BOD5), chemical oxygen demand (COD), total dissolved solids (TDS), total suspended solids (TSS), and solution pH. The BOD5 of dye wastewater samples was determined according to the ASTM D888‐18 standard using a DO HQ30d meter. The COD was assessed according to the ASTM D8084‐17 standard on a UV spectrophotometer while TDS was tested according to the ASTM D5907‐18 standard on a Handy Lab 680 EK SI Analytics (Germany). The TSS was recorded according to the ASTM D3977‐97(2017) standard, and the pH value of solutions was measured using a Mettler Toledo equipment (Switzerland). To measure COD, the dye wastewater samples were kept in flasks and placed in an oven at 150 °C for 2 hours before cooling to room temperature and measuring the COD values. For BOD measurement, distilled water as a control and dye wastewater were placed in two separate bottles and kept in an incubator at 20 °C for 5 days. Subsequently, the BOD value is determined by comparing the dissolved oxygen of the control sample and the wastewater samples. To measure TSS and TDS, the filter paper and clean glass beaker were dried and weighed before use. One liter of wastewater samples was filtrated using the above filter paper. Upon filtrating, the filter paper was dried and reweighed carefully to record the TSS value. Consequently, the filtered water is poured into a clean, dried glass beaker and placed in an oven to evaporate the water. After cooling, the beaker was reweighed to determine TDS.

## 2 Results and Discussion

### 2.1 Dyeing Capacity of Cotton Fabrics

Table 1 presents the color changes of two cotton fabric samples dyed with the DME and reactive dye. Both dyes exhibit the ability to dye cotton fabric to black color as indicated by the L* value. The L* value of cotton fabrics dyed with the reactive dye is 20.68, lighter than the cotton fabrics dyed with the DME by 3.2 %. Additionally, the color change (ΔE*) and K/S of the cotton fabrics dyed with the reactive dye is 21.52 and 19.36, respectively, differing from the cotton fabrics dyed with the DME by 14.2 % and 4.3 %, respectively. Moreover, the one‐way ANOVA and Turkey HSD tests indicate that ΔE* and K/S values of two dyed fabric samples are significantly different. This difference may be caused by the composition and chemical structure of colorants in DME and RGB.[^43^, ^44^] The main component of DME is Diospyros, a black colorant for fabrics, that can absorb and reflect the light better than RGB. In comparison with the report of Chitichotpanya *et al*., the cotton fabrics dyed with DME in this study exhibit much lower K/S as compared to the cellulosic hemp fabrics dyed with *Diospyros mollis* Griff. colorant (K/S of 9.85).^[43]^ This may be due to the difference in the geography between Vietnam and Thailand as well as in the dyeing stages and the extraction conditions.

**Table 1:** Color change of cotton fabrics dyed with DME and RGB.

Sample	L*	a*	b*	C*	H*	ΔE*	K/S	Macroscopic image
Cotton fabrics dyed with DME	20.02±0.02	3.36±0.01	4.46±0.01	5.58±0.01	52.96±0.20	25.09±0.01	18.52	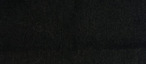
Cotton fabrics dyed with RGB	20.68±0.02	3.56±0.01	4.78±0.01	5.96±0.01	53.32±0.02	21.52±0.01	19.36	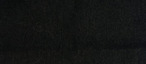
						p<0.01	p<0.01	

### 2.2 Morphology of Dyed Cotton Fabrics

**Figure 3:**
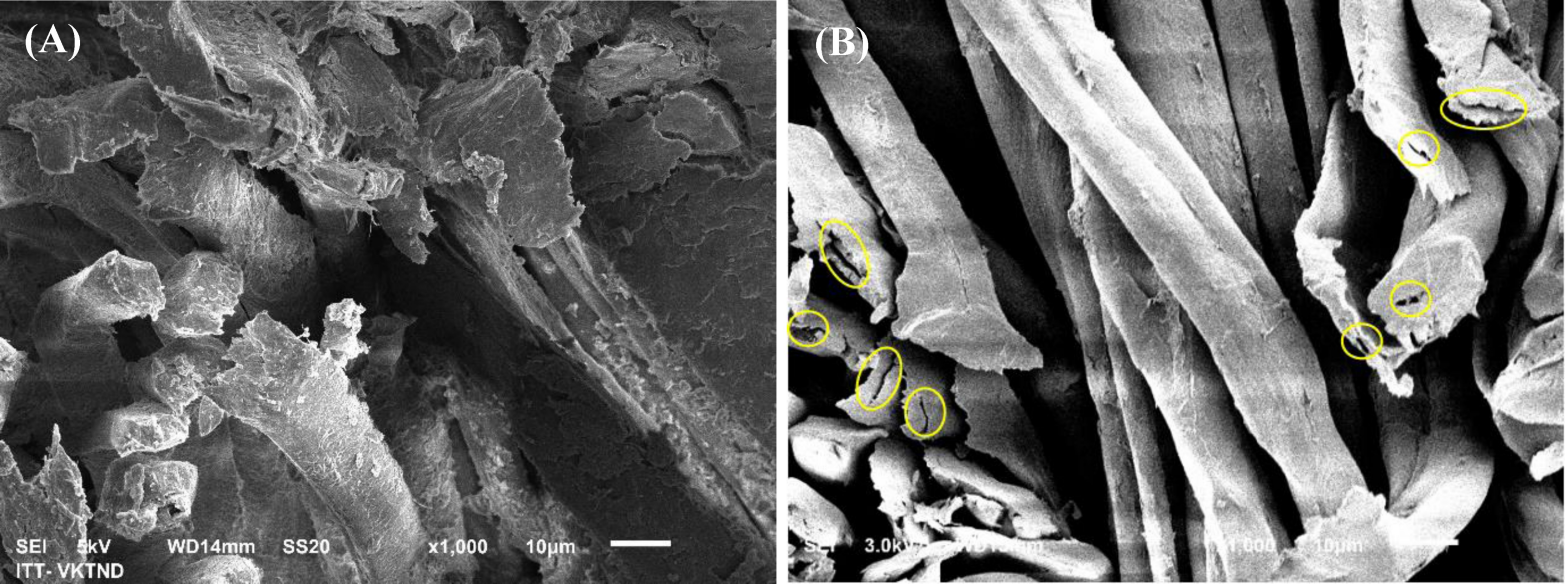
SEM images of cross‐surface of cotton fabrics dyed with DME (A) and RGB (B)

SEM images of the cross‐surface of the dyed cotton fabrics were shown in Figure 3. The difference in the cross‐surface of the cotton fabrics dyed with DME (Figure 3A) and that dyed with the RGB (Figure 3B). Both the surfaces of fibrils and cross‐surface of the cotton fabrics dyed with the RGB are smooth while the surface of cotton fibrils dyed with DME is rough caused by the adhesion and absorption of organic compounds on the surface of fibrils.^[45]^ Moreover, the cross‐surface of the cotton fabrics dyed with DME is tight, suggesting that the miscibility of DME with cotton fibrils while that of the cotton fabrics dyed with RGB exhibits empty grooves, confirming its less tight structure and the poor mixing of RGB and cotton fibrils. The appearance of empty grooves also reflects the poor ability of the cotton fabrics dyed with RGB to withstand the impact of external forces when cutting.

### 2.3 Functional Groups in Dyed Cotton Fabrics

**Figure 4:**
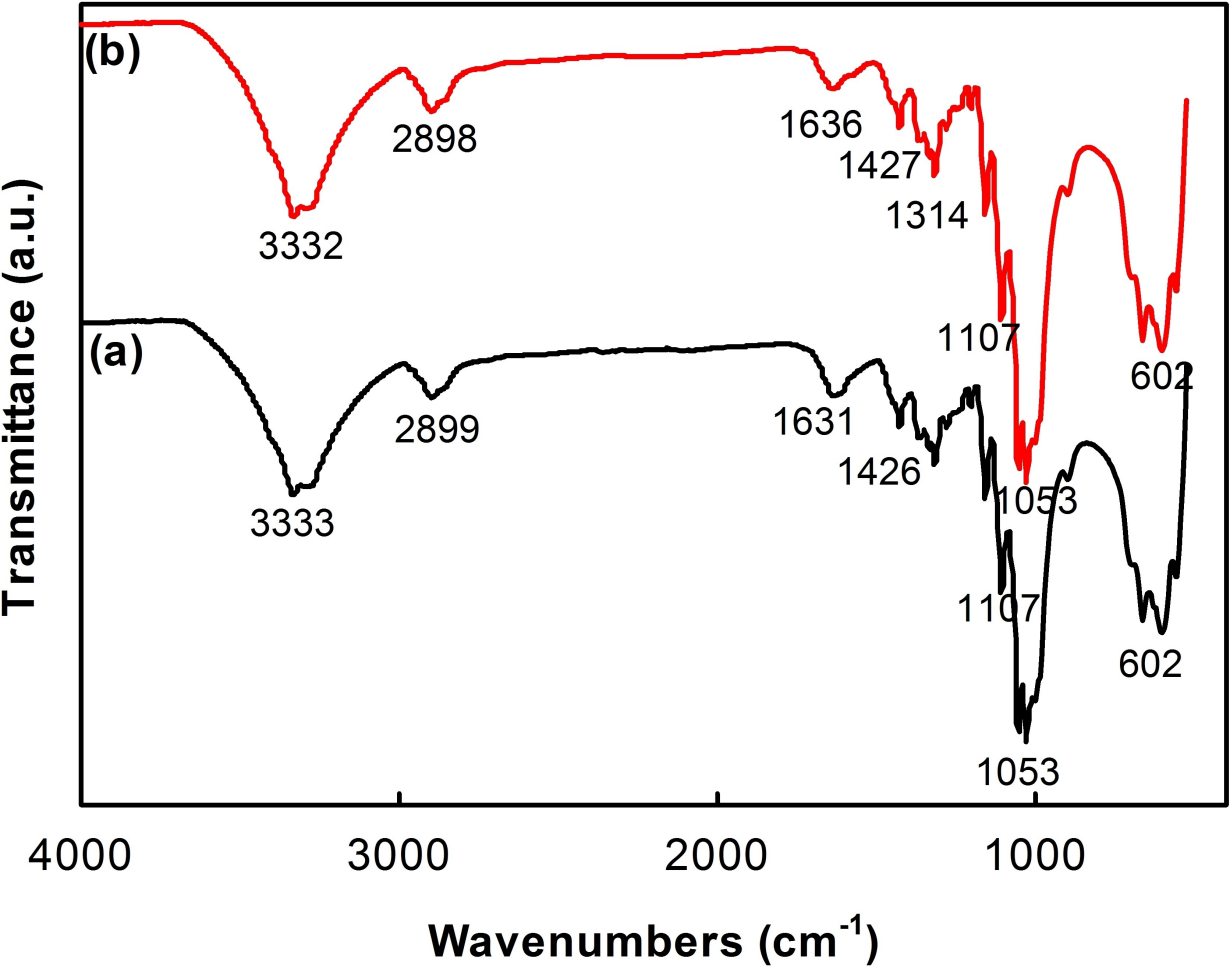
FTIR spectra of cotton fabrics dyed with DME (a) and RGB (b)

FTIR spectra of the cotton fabric samples dyed with RGB and DME are displayed in Figure 4. The peaks characterized for vibrations of cellulose appeared on two FTIR spectra of both fabric samples. For example, the stretching vibrations of O−H linkage were found at 3333 cm^−1^, the stretching vibration of C−H linkage was assigned at 2899 cm^−1^, the bending vibrations of O−H and C−H linkages were located at 1631 cm^−1^ and 1426 cm^−1^, respectively,^[46]^ the stretching vibrations of C−O, C−C bonds appeared in 1000–1200 cm^−1^.[^47^, ^48^] The characteristic peaks of cellulose in both samples are in the same location, but the small difference in the intensity of peaks on the FTIR spectra may be due to the difference in the thickness of the samples. Thickness can affect the IR radiation absorption and transmittance ability of fabric samples. It can suggest that the cotton fabrics dyed with DME exhibit a lower thickness than those dyed with RGB. Another reason may be the absorbance ability to IR radiation of organic compounds in the cotton fabrics dyed with DME is better than that in cotton fabrics dyed with RGB.^[43]^ A proposed interaction model of tannic acid in DME with cellulose through hydrogen bonds and dipole‐dipole interactions has been mentioned previously.^[43]^ The reactive dyes can interact with cellulose in an aqueous medium through hydrogen bonds, ionic bonds, and covalent bonds.^[12]^

### 2.4 Antibacterial Ability of Dyed Cotton Fabrics

**Figure 5:**
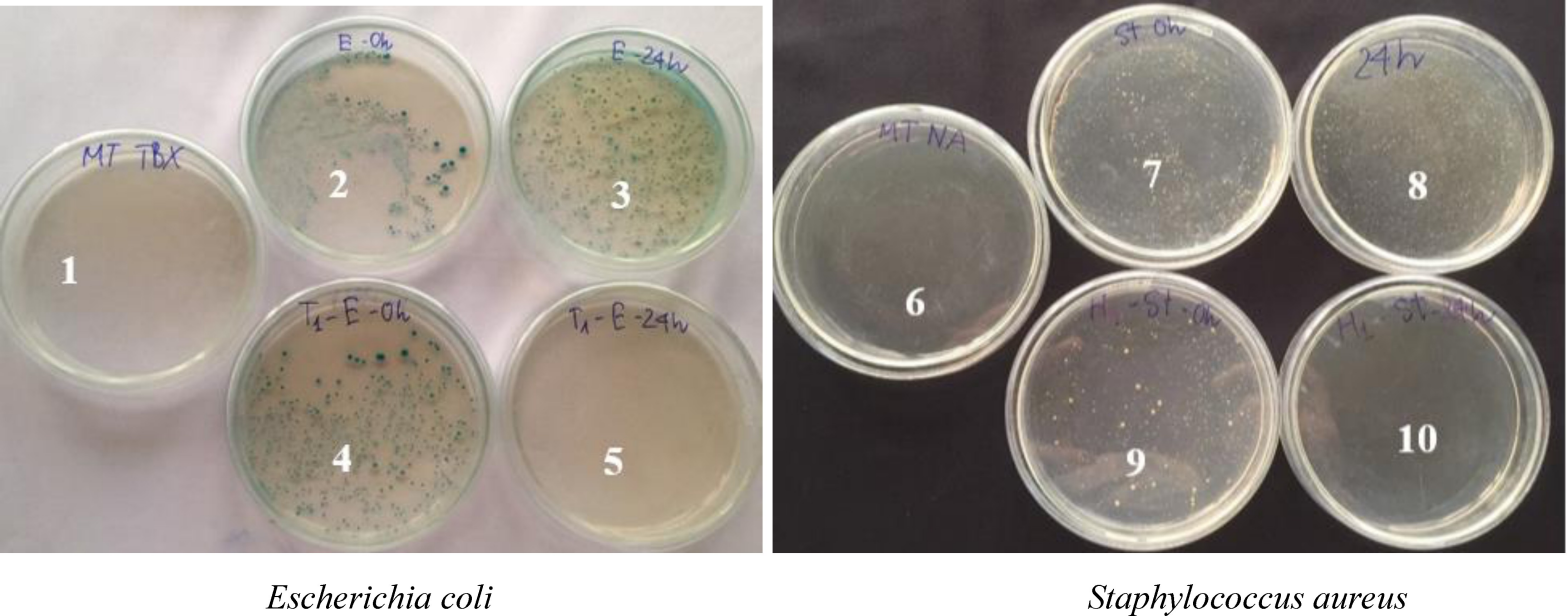
Test agar images containing cotton fabrics dyed with RGB at 0 hour (dishes 2, 7) and at 24 hours (dishes 3, 8); positive control sample at 0 hour (dishes 4, 9) and at 24 hours (dishes 5, 10); negative control sample (dishes 1, 6).

Table 2 indicates the outstanding antibacterial activity of the cotton fabrics dyed with DME as compared to those dyed with RGB. The cotton fabrics dyed with DME can kill *Escherichia coli* and *Staphylococcus aureus* with a decline percentage of 99.99 % while the cotton fabrics dyed with RGB cannot be antibacterial (Figure 5 and Table 2). The cotton fabric dyed with DME has good antibacterial properties because the DME solution contains tannin and hydroquinone which have great antibacterial ability.[^49^, ^50^, ^51^, ^52^] The antibacterial activity of the cotton fabrics dyed with DME in this study is better than that of the cellulose dyed with *Diospyros mollis* Griff. colorant (% reduction of *Escherichia coli* and *Staphylococcus aureus* reach 92.41 and 97.15 %, respectively)^[43]^ due to some reason as earlier mentioned.

**Table 2:** Antibacterial activity of cotton fabrics dyed with DME and RGB.

Bacterial strain			Result
Reduction rate of bacteria on the cotton fabrics dyed with DME	*Escherichia col* ATCC 25922	0 hour, CFU/sample	1.9×$10^{5}$
		24 hours, CFU/sample	1.6×$10^{3}$
		**% reduction**	**99.99**
	*Staphylococcus aureus* ATCC 6538	0 hour, CFU/sample	1.9×$10^{5}$
		24 hours, CFU/sample	1.2×$10^{4}$
		**% reduction**	**99.99**
Reduction rate of bacteria on the cotton fabrics dyed with RGB	*Escherichia col* ATCC 25922	0 hour, CFU/sample	1.9×$10^{5}$
		24 hours, CFU/sample	1.9×$10^{5}$
		**% reduction**	**0**
	*Staphylococcus aureus* ATCC 6538	0 hour, CFU/sample	1.9×$10^{5}$
		24 hours, CFU/sample	1.9×$10^{5}$
		**% reduction**	**0**

### 2.5 UV Resistance of Dyed Cotton Fabrics

Table 3 displays the UV resistance ability of cotton fabric dyed with DME and dyed with RGB. The UV protection ability of the fabrics is reflected through the UPF index, a rating system for apparel, indicating how effectively a fabric shields the skin from UV rays. The UPF index of the cotton fabrics dyed with DME reaches 54.08 while that of the cotton fabrics dyed with RGB reaches 36.82. According to the AS/NZS 4399 : 1996 test method, the cotton fabrics dyed with DME exhibit an excellent UV protection ability with an UPF value above 40 while the cotton fabrics dyed with RGB exhibit a very good UV protection ability with an UPF value below 39. On the other hand, UV transmittance percentage in UV regions determines the penetrability of UV rays to the skin or the associated effects. The UV protection property of a dyed fabric is considered “good” when the UV transmittance is below 5 %. In this study, the percentage transmittance values of UV‐A (315 nm to 400 nm) and UV‐B (290 nm to 315 nm) radiation of cotton fabrics dyed with DME are lower than 0.1 % and that of cotton fabrics dyed with RGB is below 2.05 %. The results show a significant difference between the UV protection ability of the cotton fabrics dyed with DME and that of the cotton fabrics dyed with RGB (p‐value <0.01). The UV protection ability of the cotton fabrics dyed with DME is better than that of the cotton fabrics dyed with RGB by 31.92 %. The cotton fabrics dyed with DME exhibit excellent UV shielding properties. Although the RGB can absorb and reflect UV rays,[^53^, ^54^] it cannot completely cover the cotton fibrils’ surface after dyeing as shown in SEM images, allowing more UV‐A and UV‐B rays to pass through the cotton fabrics. In contrast, for the cotton fabrics dyed with DME, the organic compounds cover the surface of cotton fibrils, helping to limit UV ray transmittance due to the UV absorbance ability of polyphenolic compounds.^[55]^ Furthermore, tannin – the main component in the DME can act as a UV shield when adhesion on the surface of cotton fibrils.^[56]^ The cotton fabrics dyed with DME in this study exhibit outstanding effectiveness in their properties, antibacterial ability, and UV resistance ability as compared to the cellulose dyed with DME.^[43]^ This indicates the promise of the cotton fabrics dyed with DME in this study for various applications, especially in UV protection and antibacterial textiles.

**Table 3:** UV resistance ability of dyed cotton fabrics.

Sample	UPF	UV‐A Transmittance (%)	UV‐B Transmittance (%)
Cotton fabrics dyed with DME	54.08	0.08	0.05
Cotton fabrics dyed with RGB	36.82	2.03	1.21
	p<0.01	p <0.01	p<0.01

From the obtained results, we can propose a mechanism to protect against UV‐A and UV‐B rays of the cotton fabrics dyed with DME and RGB dyes. Organic compounds and inorganic compounds diffuse from the outer layer of the cotton fabric then went deep into the core of cotton fibers and were retained in the fabrics. Therefore, the ability to reflect and absorb UV rays of the cotton fabrics after dyeing is effective. The absorbance ability of organic compounds in dyes allows them to effectively block UV rays while simultaneously coloring.^[43]^

### 2.6 Air Permeability of Dyed Cotton Fabrics

The air permeability of the cotton fabrics dyed with DME and the cotton fabrics dyed with RGB is demonstrated in Table 4 with values of 21.1 L/m^2^.s and 22.2 L/m^2^.s, respectively. The air permeability of cotton fabrics dyed with DME is reduced by 4.95 % compared to the cotton fabrics dyed with RGB. This reduction can be explained by the organic molecules in the DME solution going deep into the capillaries of cotton fibers, into the interlayer area between the cotton fibers, and filling the gaps of the textile fibers in the fabric. The reactive dye molecules penetrate deep into the capillaries of the cotton fibers but do not fill the gaps of the textile fibers in the cotton fabric.^[57]^

**Table 4:** Some characteristics and mechanical properties of dyed cotton fabrics.

Characteristics	Cotton fabrics dyed with DME	Cotton fabrics dyed with RGB	% Change	p‐value
Air permeability (L/m^2^.s)	21.10±0.10	22.20±0.04	−4.95	<0.01
Water vapor absorption (%)	17.23±0.01	16.62±0.03	3.67	<0.01
Cotton fabric weight (g/m^2^)	2.22±0.00	1.99±0.01	11.56	<0.01
Tensile strength in warp (Nm)	667.67±0.47	561.00±0.82	19.01	<0.01
Tensile strength in weft (Nm)	435.67±0.47	342.00±0.82	27.39	<0.01
Elongation at break in warp (mm)	69.22±0.94	58.36±0.03	18.61	<0.01
Elongation at break in weft (mm)	45.13±0.04	38.20±0.02	18.15	<0.01

### 2.7 Water Vapor Absorption and Weight of Dyed Cotton Fabrics

The data in Table 4 present the increase in the water vapor absorption and weight of the cotton fabrics dyed with DME by 3.67 % and 11.56 %, respectively, as compared to that of the cotton fabrics dyed with RGB. This increase may be due to the difference in the composition of the two dyes as aforementioned. Another reason is the microstructure of the cotton fabrics dyed with DME is tighter than that of the cotton fabrics dyed with RGB as shown in SEM images. On the other hand, organic molecules such as saponins, tannins, and hydroquinone on the surface of cotton fibrils can interact with water molecules thanks to their polarity and hydrophilic properties,[^58^, ^59^] leading to a higher water vapor absorption of the cotton fabrics dyed with DME.

### 2.8 Tensile Properties of Dyed Cotton Fabrics

The tensile strength and elongation at break of cotton fabrics dyed with DME and RGB in Table 4 exhibit a difference significantly with a p‐value <0.01. The tensile strength and elongation at the break of the dyed cotton fabrics in the warp direction are higher than that in the weft direction. The difference in tensile strength of the cotton fabrics dyed with DME as compared to the cotton fabrics dyed with RGB is larger than that in elongation at break. The cotton fabrics dyed with DME exhibit a better tensile property than the cotton fabrics dyed with RGB. This may be due to the hydroxyl groups in tannins and *Diospyros* in the DME binding to the hydroxyl groups of the cotton fabric through hydrogen bonds and van der Wall forces,^[60]^ leading to a tight microstructure and a better ability to withstand the impact of external forces. As a result, the tensile property of the cotton fabrics dyed with DME is enhanced. For RGB reactive dye, although it can form a covalent bond between the active groups of the dye and the hydroxyl groups of the cellulose fibers, the permanent binding capacity of reactive dyes for cotton fabric is about 60–70 %,^[61]^ leading to a lower tensile property of the cotton fabrics dyed with RGB as compared to that dyed with DME.

### 2.9 Color Fastness to Washing, Rubbing, and Light of Dyed Cotton Fabrics

The data of color fastness to washing, rubbing, and light of the dyed cotton fabrics in Table 5 show the color fastness to washing of the cotton fabrics dyed with DME is lower than that of the cotton fabrics dyed with RGB while their color fastness to rubbing is equal. In contrast, the color fastness to light of the cotton fabrics dyed with DME is better than that of the cotton fabrics dyed with RGB. The color fastness to washing of the cotton fabrics dyed with DME only reached level 3 after 30 washing cycles due to partly organic compounds being removed under the impact of the soap. The moderate rubbing color fastness of the cotton fabrics dyed with DME and RGB may be due to the strong impact of white spirit solvent on the bond of dyes and cotton fibers.^[62]^ The color fastness to light of cotton fabric dyed with DME is higher than that of fabric dyed with RGB 2‐level. This may be due to Diospyros continuing to turn black under the influence of air oxygen and light radiation,^[63]^ therefore, the cotton fabrics dyed with DME have high light fastness. The light color fastness of the cotton fabrics dyed with RGB depends on the organic chromophore of the reactive dye. The azo chromophore group has low light color fastness, so under the impact of light,^[64]^ the light color fastness of the cotton fabrics dyed with RGB will be reduced.

**Table 5:** Color fastness to washing, rubbing and light of dyed cotton fabrics.

Sample	Color fastness to washing				Color fastness to rubbing	Color fastness to light
	Number of washings					
	1	10	20	30		
Cotton fabrics dyed with RGB	5	4	4	4	4	5
Cotton fabrics dyed with the DME	5	4	3‐4	3	4	7

### 2.10 Parameters of Wastewater After Dyeing

Table 6 demonstrates the results of the dye wastewater discharged from dyeing process of cotton fabrics with RGB and DME (abbreviated as RGB wastewater and DME wastewater, respectively). The BOD_5_, COD, TDS, TSS and pH of the RGB wastewater and the BOD_5_, COD of the DME wastewater are higher than parameters required in the DOE standard. However, the TDS, TSS and pH of the DME wastewater meet the requirements of DOE standard, especially, the BOD_5_ of DME wastewater is much lower than that of RGB wastewater. The ratio of BOD_5_/COD (B/C) could be referred to evaluate the ability to treat textile wastewater. The B/C is higher, the treatment of dye wastewater is easier.[^65^, ^66^] Based on that, the B/C of the DME wastewater reaches 0.70 while that of the RGB wastewater reaches 0.32. This suggests that the DME wastewater can be treated more easily than the RGB wastewater. This can be due to the azo chromophore molecules in RGB active dye making it more difficult for microorganisms to decompose them.^[67]^ Moreover, the high value of COD in RGB wastewater is also caused by the dye residues after dyeing.

**Table 6:** Dye wastewater parameters.

Parameters	The wastewater discharged from dyeing process of cotton fabrics with RGB (mg/L)	The wastewater discharged from dyeing process of cotton fabrics with DME (mg/L)	QCVN 13‐MT : 2015/BTNMT (DOE) (mg/L)
BOD_5_	1258	586	50
COD	1786	1809	200
TDS	2350	1960	2100
TSS	279	146	150
pH	9.5	6	5.5–9

The TSS of DME wastewater is much lower than that of RGB wastewater. It can be explained by the dissolution of DME in water is better than that of RGB.^[68]^ The high TDS value of RGB wastewater is due to the use of Na_2_SO_3_ and auxiliaries in the last stage of dyeing process.^[69]^

DME wastewater consists of organic compound residues that can be biodegraded, so the biological treatment process of wastewater containing DME dye is often used because of its simple operation, flexibility, low cost, adaptable to the environment, environmentally friendly, and great COD removal ability.[^70^, ^71^, ^72^] However, for RGB wastewater, azo ring compounds are difficult to break down due to microbial decomposition. Consequently, it requires adding the supplement chemicals in the wastewater treatment process.[^73^, ^74^, ^75^, ^76^] This leads to complicated wastewater treatment and increased costs. For DME wastewater, we propose the treatment plan with some steps as follows: (i) the DME wastewater is transferred to the sedimentation tank to get the water and sludge parts. The wastewater part is treated with microorganisms, the sludge is processed into organic fertilizer to fertilize plants.

The new findings in this study give a panoramic picture of the dyeing efficiency of the aqueous *Diospyros mollis* Griff extract, a natural dye, compared to the Remazol deep black RGB active dye, a synthetic dye. The evidence confirms the superiority of natural dye over synthetic dye for cotton fabrics with excellent antibacterial ability, good UV protection, and high mechanical properties. Additionally, the ability to treat wastewater from the dyeing process by using microorganisms also proves the potential of natural dyes.

## 3 Conclusions

The aqueous *Diospyros mollis* Griff extract (DME) has shown good dyeing capacity, high color fastness to washing, light, and rubbing, antibacterial ability against *E. coli* and *S. aureus* 99.9 %, great UV resistance efficiency, good air permeability, water vapor absorbance, a high tensile property when was applied for dyeing the cotton fabrics as compared to the Remazol deep black RGB active dye. The DME can interact well with the hydroxyl groups on the surface of cellulose fibers, filling the space in the fibers to form a tight microstructure for the dyed cotton fabrics. The wastewater discharged from the dyeing process of cotton fabrics with DME is easier to treat than that with RGB active dye. From the obtained results, it was found that the cotton fabrics dyed with DME have many good advantages, bringing high value to textile products, taking advantage of available natural raw materials, and having little impact on the environment. DME natural dye can be used as a substitute for reactive dyes when dyeing cotton fabric black. Further studies can develop modern dyeing technology such as ultrasonic dyeing, UV irradiation, etc. for various types of fabrics with DME. Moreover, the technology and optimal conditions for the treatment of dyeing wastewater are also focused on the research to minimize environmental pollution caused by fabric dyeing.

## Author Contributions

All authors contributed to the study conception and design. Material preparation, data collection, and analysis were performed by Trong Tuan Nguyen, Thuy Chinh Nguyen, Thi Thu Trang Nguyen, Ha Nguyen Manh, and Hoang Thai. The first draft of the manuscript was written by Trong Tuan Nguyen and all authors commented on previous versions of the manuscript. All authors read and approved the final manuscript.

## Conflict of Interests

The authors have no relevant financial or non‐financial interests to disclose.

4

## Data Availability

The datasets used and/or analyzed during the current study are included in the article.
